# Severe acute pancreatitis with blood infection by *Candida glabrata* complicated severe agranulocytosis: a case report

**DOI:** 10.1186/s12879-018-3623-6

**Published:** 2018-12-29

**Authors:** Rong Shi, Qianmei Zhou, Rong Fang, Xudong Xiong, Qian Wang

**Affiliations:** 1 Department of Emergency Internal Medicine, Shuguang Hospital Affiliated to Shanghai University of Traditional Chinese Medicine, 528 Zhangheng Road, Pudong New Area, Shanghai, 201203 People’s Republic of China; 2 Research Center for Traditional Chinese Medicine Complexity System, Shanghai University of Traditional Chinese Medicine, Shanghai, 201203 People’s Republic of China

**Keywords:** Blood infection, Candida Glabrata, Severe acute pancreatitis

## Abstract

**Background:**

Blood infection with Candida glabrata often occurs in during severe acute pancreatitis (SAP). It complicate severe agranulocytosis has not been reported.

**Case presentation:**

We present a case where a SAP patient presenting with a sudden hyperpyrexia was treated for 19 days. We monitored her routine blood panel and CRP levels once or twice daily. The results showed that WBC count decreased gradually. And the lowest level of WBC was appeared at the 21st day of treatment. WBC 0.58 × 10^9^/L(4.0–10.0 × 10^9^/L), neutrophils 0.1 × 10^9^/L [2.20%] (2.5–7.5 × 10^9^/L). During treatment, *Candida glabrata* was identified as the infecting agent through blood culture, drainage tubes culture and gene detection. During anti-infection therapy, the patient had severe agranulocytosis. With control of the infection, her WBC and granulocyte counts gradually returned to the normal range.

**Conclusions:**

Blood infection with Candida glabrata can complicate severe agranulocytosis.

## Background

Acute pancreatitis (AP) is an acute inflammatory reaction of the pancreas. Most of AP can be healed by themselves. Approximately 20% of AP cases progress to severe AP (SAP). SAP mortality remains high and clinical diagnosis and treatment continue to be considerable challenge [1, 2]. Numerous diseases and symptoms, such as bacteraemia, a high Ranson score, and diabetes, can be significantly associated with mortality in SAP patients [3–5]. Infection is a common clinical complication in the latter stages of SAP, and blood infection from *Candida glabrata* often occurs in such patients. However, complication with severe agranulocytosis has not been reported.

This paper presents a case of a SAP patient who presented with a sudden hyperpyrexia and chills and was treated for 19 days. Blood culture and high-throughput gene detection indicated *C. glabrata* infection. On the 20th day of treatment, the patient experienced sudden agranulocytosis. She subsequently recovered after active anti-infection and symptomatic treatment for 11 days. This case is reported as follows.

## Case presentation

After 5 h of abdominal distention and pain, a 26-year-old Chinese woman reported hospital at 15:30 on December 3, 2017. The patient had previously been hospitalised for AP due to hyperlipidaemia on May 9, 2017, after which she had discontinued the lipid-lowering drugs prescribed by her doctor. During the 3 months before her admission in December, she resumed a high-fat diet. Approximately 7 h before disease onset, the patient consumed fatty food even after the occurrence of abdominal distention and pain. Her abdominal pain gradually worsened, and she vomited twice. The patient was diagnosed with AP based on her medical history, symptoms, signs, hemodlastase, and upper abdominal computed tomography (CT). After 10 h of hospitalisation, her abdominal pain became aggravated, leading to haemodynamic instability. Upper abdominal CT, *liver*, *kidney*, and *heart* function and electrolyte levels were reviewed. A comprehensive evaluation of the patient’s condition revealed a Ranson score of 4, Balthazar CT grade of D, APACHE II score of 17, and SOFA score of 9. The patient was diagnosed with SAP and multiple organ dysfunction syndrome *(heart*, *liver* and *kidney*). After hospital admission, the patient was treated with positive expansion, gastrointestinal decompression, and nutritional support, and continuous renal replacement therapy (CRRT) treatment was initiated on the second day. Based on an examination of abdominal imaging, intraperitoneal puncture and drainage was administered under the guidance of ultrasound on days 2, 4, 8, and 15. Subsequently, eight root drainage tubes were placed (pull out of the two tubes of the eight tubes on the 11th day) and jejunal nutrition was administered for 16 days after admission. By day 18 after admission, the patient’s renal function had restored, and intraperitoneal pressure had decreased from 32 mmHg at admission to 13 mmHg. The APACHE II and SOFA scores both became 3 on day 18. Onday 19, the patient’s temperature was within the healthy range at 06:00. The results of a routine blood examination were as follows: white blood cells (WBCs) 9.61 × 10^9^/L, neutrophils 7.8 × 10^9^/L (81%), C-reactive protein (CRP) 39.61 mg/L. Until 17:00 on day 19, the patient experienced chills and high fever. Her body temperature reached a high of 40.2 °C. After blood culture, linezolid and meropenem were performed to anti-infection treatment immediately. Routine blood examination, procalcitonin (PCT), and CRP levels were also observed (WBCs4.64 × 10^9^/L, neutrophils 3.4 × 10^9^/L [73.6%], CRP 51.15 mg/L, PCT 2.68 ng/mL). The patient continued to experience high fever on day 20. Thus, we administered a deep vein puncture tube and removed the remaining six root abdominal drainage tubes. All extracted peritoneal drainage tubes were used to make normalism the patient’s etiological cultivation. The causative agents were determined through high-throughput gene detection from a venous blood sample. Blood culture was performed again. These tests revealed that the patient had a fungal blood infection. Based on the conventional treatmet method, caspofungin was added to the drug regimen. On the 23rd day of treatment, Tc*. glabrata* was identified as the infecting agent through blood culture and gene detection.

Eventually, we discontinued the administration of meropenem and linezolid, but continued that of caspofungin. The patient’s body temperature was restored to within the normal range on the 25th day of treatment. During treatment, we monitored her routine blood panel and CRP levels once or twice daily. The results showed that her WBC count decreased gradually—to its lowest level on the 21st day of treatment (WBCs 0.58 × 10^9^/L, neutrophils 0.1 × 10^9^/L [2.20%]). Her haemoglobin and platelet levels also decreased. However, the duration of this decline was shorter than that of the WBC decline. The levels of other inflammatory markers also increased on the 23rd day of treatment (CRP 235.89 mg/L, PCT 10.85 ng/mL), alongside an increase in creatinine levels. On the 32nd day of treatment, the patient’s WBC count was finally restored to the normal range.

In summary, the patient’s temperature returned to normal with caspofungin treatment for 10 days. Blood culture was carried out on the 26th and 30th days after admission, and no bacterial growth was found in the two blood cultures. She was discharged on the 33rd day after treatment. And she was followed up every 10 days for 30 days after discharge. Results showed that her temperature was normal. Since then, she has been maintaining the low fat diet.

**Fig. 1:**
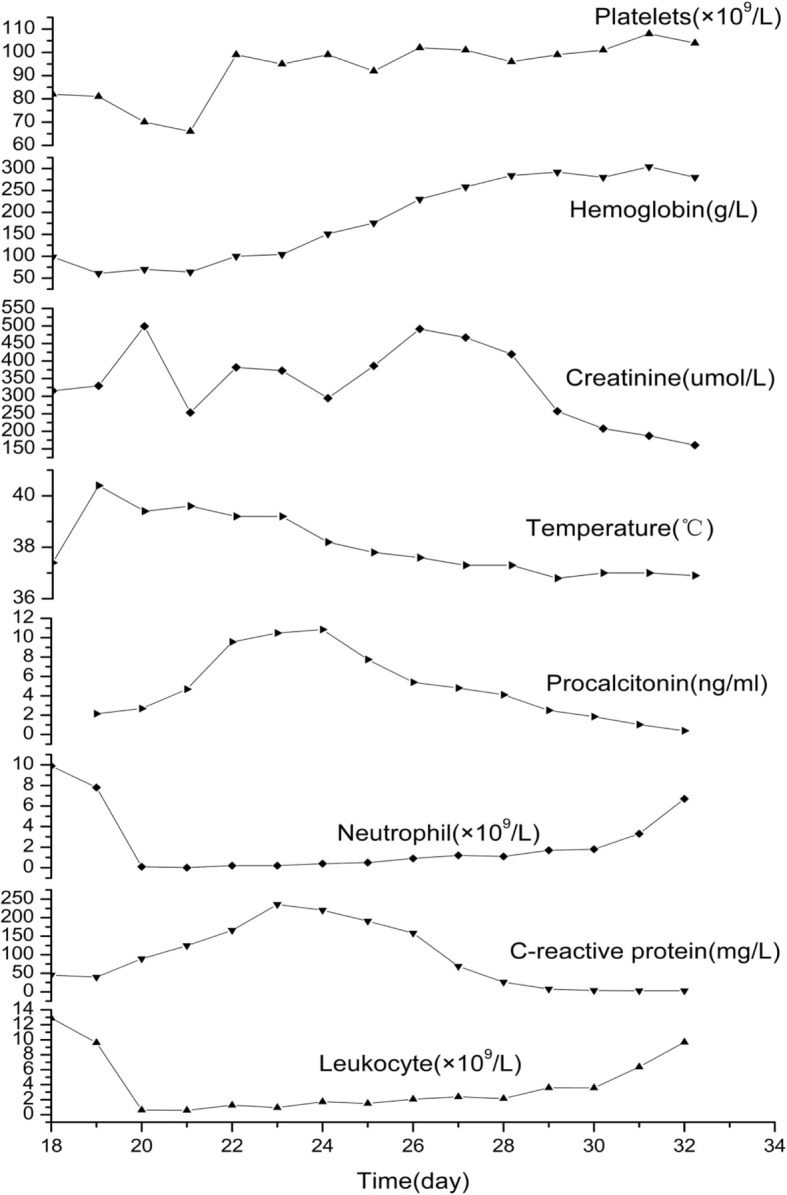
Variable trend of blood values

The timings of the observations (WBC counts, neutrophils, CRP, PCT, ect.) are shown in Fig. 1.

## Discussion and conclusions

With the aging of hospitalized patients and the coexistence of many basic diseases, multiple invasive diagnostic procedures, such as broad-spectrum antibiotics, glucocorticoids, immunosuppressive agents, chemotherapy drugs, central venous catheterization and hemodialysis are widly performed [6, 7]. Therefore, incidence of acquired candidiasis and the associated mortality rate have increased [8, 9]. Because of its lack of specific clinical manifestations, candidaemia cannot be diagnosed early, and thus, its mortality rate is high [10].

*C. glabrata* is typically a nonpathogenic symbiotic bacteria in the human mucosa, and causes opportunistic infections only occasionally. In recent years, the incidence of blood infections due to *C. glabrata* has increased [11]. Candidaemia often occurs in patients with neutropaenia. Fw cases of severe agranulocytosis caused by *Candida* blood infection have been recorded.

Our patient was admitted to hospital for SAP. During treatment, we performed puncture and drainage under the guidance of ultrasound to relieve the intraperitoneal exudation. Although active puncture can effectively relieve intestinal injury caused by SAP [12], repeated invasive surgery and other catheter implantations increased the risk of blood infection due to *Candida* [13]. On the 19th day of treatment, the patient appeared to have sudden chills and hyperpyrexia. Considering the possibility of candidaemia, we preserved the specimen and removed the catheter. During this time, the patient was treated with caspofungin. She was subsequently diagnosed with *C. glabrata* infection based on blood culture, central venous catheterisation, abdominal puncture drainage tube culture, and high-throughput gene detection. Before *C. glabrata* infection, our patient’s WBC and neutrophil counts can be high or within the normal range. During anti-infection therapy, our patient exhibited severe agranulocytosis. After the infection had been controlled, her WBC and granulocyte counts gradually returned to within the normal range. The potential impact factors have three aspects. Firstly, some antibiotics may cause agranulocytosis, such as Linezolid and meropenem. However, in this case, the WBC count has decreased before linezolid and meropenem is performed. Therefore, we conclude that WBC decline is not directly related to the use of the above two antibiotics, but related to the blood infection caused by *C. glabrata*. Sencondly, When the patient developed shivering and high fever, she was mainly administrated with nutritional support. Drugs used in nutritional support do not cause agranulocytosis. Thirdly, during the treatment, we cultured the patient’s blood and puncture drainage tubes. There were no pathogenic bacteria associated with agranulocytosis grown. Moreover, she has no medical history of agranulocytosis. After careful exclusion other potential factors causing agranulocytosis, we concluded that her agranulocytosis was related to blood infection due to *C. glabrata*.

## Abbreviations

AP: Acute pancreatitis
CRP: C-reactive protein
CRRT: Continuous renal replacement therapy
CT: Computed tomography
PCT: Procalcitonin
SAP: Severe AP
WBC: White blood cells
